# Molecular mechanistic associations of human diseases

**DOI:** 10.1186/1752-0509-4-124

**Published:** 2010-09-06

**Authors:** Philip Stegmaier, Mathias Krull, Nico Voss, Alexander E Kel, Edgar Wingender

**Affiliations:** 1 BIOBASE GmbH, Halchtersche Strasse 33, D-38304 Wolfenbüttel, Germany; 2 Institute of Chemical Biology and Fundamental Medicine, Lavrentiev Ave.8, 630090, Novosibirsk, Russia; 3 Department of Bioinformatics, Medical School, Georg August University Göttingen, Goldschmidtstrasse 1, D-37077 Göttingen, Germany

## Abstract

**Background:**

The study of relationships between human diseases provides new possibilities for biomedical research. Recent achievements on human genetic diseases have stimulated interest to derive methods to identify disease associations in order to gain further insight into the network of human diseases and to predict disease genes.

**Results:**

Using about 10000 manually collected causal disease/gene associations, we developed a statistical approach to infer meaningful associations between human morbidities. The derived method clustered cardiometabolic and endocrine disorders, immune system-related diseases, solid tissue neoplasms and neurodegenerative pathologies into prominent disease groups. Analysis of biological functions confirmed characteristic features of corresponding disease clusters. Inference of disease associations was further employed as a starting point for prediction of disease genes. Efforts were made to underpin the validity of results by relevant literature evidence. Interestingly, many inferred disease relationships correspond to known clinical associations and comorbidities, and several predicted disease genes were subjects of therapeutic target research.

**Conclusions:**

Causal molecular mechanisms present a unifying principle to derive methods for disease classification, analysis of clinical disorder associations, and prediction of disease genes. According to the definition of causal disease genes applied in this study, these results are not restricted to genetic disease/gene relationships. This may be particularly useful for the study of long-term or chronic illnesses, where pathological derangement due to environmental or as part of sequel conditions is of importance and may not be fully explained by genetic background.

## Background

Diseases and accompanying symptoms are spawned by systems of molecules, which operate within and across cell and tissue boundaries. A major goal of medical research is to identify the molecular components which play a role in causing a pathological condition. Since first seminal achievements [1], events at the molecular level have been recognized as key to understand disease mechanisms.

Phenotype/genotype associations provide evidence for a role of affected gene products in respective causal mechanisms and extensive resources document medically relevant gene variants [2,3]. Recent studies on hereditary phenotypes have shown that similarities among disorders imply involvement of functionally related gene products, summarized as "phenotypic overlap implies genetic overlap". The modular nature of human genetic diseases suggests that modules of similar disorders, also denoted as disease subnetworks, can be juxtaposed with modules of molecules which commonly contribute to a biological function, or interact in molecular complexes or pathways [4-7]. Several studies support the modularity concept and it was successfully applied to derive computational approaches for prediction of candidate genes as well as functional links between molecules [8-12].

It is now clear that analysis of disease relationships unfolds new opportunities for both medical and biological research. Several aforementioned works determined pairwise disorder similarity with a score derived from text-mining of OMIM phenotype descriptions [5]. Rzhetsky et al. [9] analyzed associations among 161 diseases based on their co-occurrence in patient records. Possibilities to correlate diseases through protein interaction networks or molecular pathways were also explored [13,14]. Sam et al. [13] used relations between proteins, Gene Ontology (GO) [15], and phenotypes established in the PhenoGO NLP system [16] together with Reactome [17] protein interactions to find diseases involving common protein-protein interaction networks such as xeroderma pigmentosum and Cockayne syndrome, for which a functional link was previously discussed [18]. Li and Agarwal [14] obtained disease/gene associations through literature mining of MEDLINE abstracts and constructed a network of diseases which share common molecular pathways. In this network they identified novel disease relationships and observed that a disease is linked to several pathways and a pathway is linked to several diseases.

We present a novel approach to analyze mechanistic relationships between human diseases. Using about 10000 causal disease/gene associations annotated in the BIOBASE Knowledge Library (BKL) [19] a statistical method that quantifies pairwise similarity between disorders was developed. Connecting diseases at a certain significance threshold, the statistical approach revealed groups of diseases which feature characteristic biological functions. So far, computationally inferred disease relationships were mainly examined with regard to shared molecular networks. Yet, many disease associations reported in this work correspond to known clinical associations and causal links between pathologies. Furthermore, we used disease associations and gene associations to predict causal disease genes. The results suggest that analysis of causal mechanisms provides a unified framework for disease classification, discovery of causal components, and can be used to obtain computational evidence for clinical disease associations as well as hypotheses about their molecular foundation.

## Results

### A molecular mechanistic map of human diseases

We extracted disease/gene associations which had been manually classified as causal or preventative from the BIOBASE Knowledge Library™ (Methods). In the following, we denote respective genes as *causal genes*. The data set comprised 375 diseases which were connected to at least 5 of 3051 causal genes by a total of 9871 disease/gene associations. Similarity of involved molecular mechanisms for each disease pair was assessed by calculating the number of common causal genes and the corresponding P-value as described in Methods.

We first constructed a map connecting all diseases with a minimum of two common genes and a maximal similarity P-value of 0.001. This map consisted of one giant component with 123 disease nodes, three medium-sized components with 14, 12, and 10 nodes as well as 29 small components with two to six nodes. In total, there were 239 of the 375 diseases, so that 136 diseases were not connected to any other at the required similarity threshold.

We tested whether the number of 239 diseases connected at the chosen P-value threshold was statistically significant. For this, we calculated false discovery rates (FDR) for P-values of disease pairs with at least two common causal genes using the fdrtool package [20]. According to fdrtool, the P-value cut-off 0.001 corresponded to a false discovery rate of 0.024. Hence, the disease connections were statistically significant also after multiple testing correction. For comparison, 282 disorders were connected at a FDR of 0.05 (P-value 3.73e-3).

**Figure 1:**
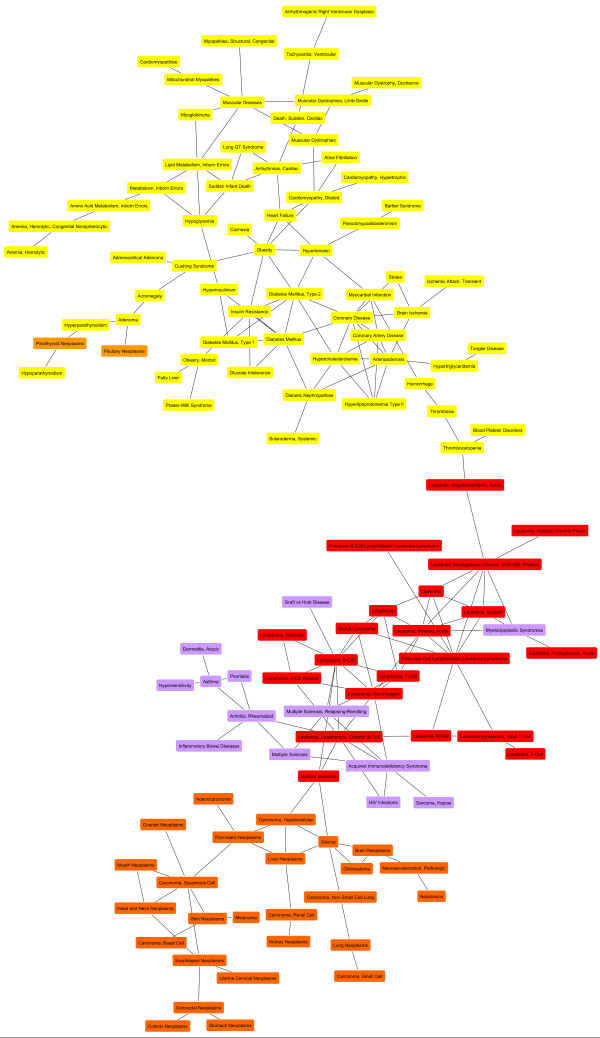
**Giant component of the human disease map**. The giant component of the disease map comprised 123 disease nodes (yellow, orange, red, and purple nodes) arranged in three subregions. Some disease names are highlighted. The top region (yellow and orange nodes) contains muscular, cardiovascular and metabolic disorders as well as parathyroid neoplasms and pituitary neoplasms. The middle region is made up of hematological malignancies (red nodes) and immune system-related pathologies such as multiple sclerosis, acquired immunodeficiency syndrome, and rheumatoid arthritis (purple nodes), whereas non-hematological malignancies are located in the lower region (orange nodes).

**Figure 2:**
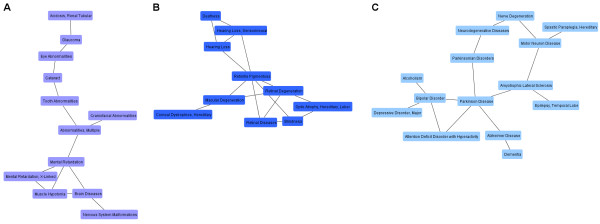
**Medium-sized components of the human disease map**. The figure shows three medium-sized components of the disease map with some disease names highlighted. One group (A) comprises variants of congenital mental retardation, eye abnormalities, tooth abnormalities as well as glaucoma, cataract and renal tubular acidosis. Retinal diseases, blindness as well as hearing loss and deafness are joined in another group (B). The third disease group (C) contains Parkinsonian disorders, Alzheimer disease, dementia, as well as bipolar disorder and alcoholism.

Fig. 1 and Fig. 2 show the giant component and the three medium-sized components.

In the giant component, diseases are congregated in three subregions. The top of the network (yellow colored, Fig. 1) comprises mostly muscular, cardiovascular and metabolic diseases such as diabetic disorders, obesity, myopathies and heart failure, but also stroke and brain ischemia. Many of the disease entities gathered in this region are recognizable as components of the cardiometabolic syndrome, a clinical clustering of cardiovascular disease risk factors like obesity, hypertension, and insulin resistance [21,22]. Notably, two neoplastic diseases, namely parathyroid neoplasms and pituitary neoplasms (orange nodes), are located in a branch shared with acromegaly, adenoma, hyperparathyroidism, and hypoparathyroidism (yellow nodes). Acromegaly is an endocrine disorder which is caused in more than 95% of the cases by benign, growth hormone producing pituitary adenoma [23]. Other endocrine neoplasia such as parathyroid neoplasms can occur as part of an acromegaly-causing syndrome called multiple endocrine neoplasia (MEN) [24]. Hence, this branch involves endocrine disorders and known comorbidities.

Through thrombosis and thrombocytopenia, also connected to the more general class of blood platelet disorders, the top region is joined with an area containing hematological malignancies like leukemia and lymphoma (red nodes, Fig. 1) as well as several other immune system-related pathologies among others multiple sclerosis, acquired immunodeficiency syndrome, and rheumatoid arthritis (purple nodes, Fig. 1). The third subregion of the giant component contains exclusively non-hematological malignancies like liver neoplasms, brain neoplasms, and melanoma (orange colored, Fig. 1). The connection to the central part of the component occupied by immune system-related disorders occurs through hepatocellular carcinoma and glioma, which are linked with multiple myeloma.

The three medium-sized components (Fig. 2) represent developmental abnormalities, audio-visual disorders as well as neurodegenerative and psychiatric illnesses. One cluster (Fig. 2A) concatenates, among others, variants of congenital mental retardation, eye abnormalities, tooth abnormalities as well as glaucoma, cataract and renal tubular acidosis. Retinal diseases, blindness as well as hearing loss and deafness are located together in another group (Fig. 2B). In the third disease group (Fig. 2C), we find Parkinsonian disorders, Alzheimer disease, dementia, as well as bipolar disorder and alcoholism. Several of the smaller disease groups (Additional file 1) reflect the hierarchy of MeSH headings which are used for BKL disease annotation, e.g. hepatitis descriptors (group 7), ataxias (group 14), osteoporosis and postmenopausal osteoporosis (group 21), and growth disorders and dwarfism (group 31). The link between xeroderma pigmentosum and Cockayne syndrome (group 10) as well as their connection to the hair disease Trichothiodystrophy were previously discussed [13,18,25,26].

To examine whether the revealed disease associations reflect common causal mechanisms, we compared GO assignments of genes in the six largest disease groups. The Gene Ontology [15] is an extensive resource of functional annotations of genes in three main categories Biological Process, Molecular Function and Cellular Component. The Fisher test is typically used to test for significant enrichment of GO categories in gene sets (Methods). Starting from enrichment P-values obtained with the standard test, we assigned GO biological processes to disease groups identified in this work and ranked them by a preponderance value that compares P-values of different gene sets (Methods). Beyond identification of significantly enriched biological functions, this comparative approach enables to detect functional differences between gene sets even when the standard method does not assign top ranks to respective GO categories. The analysis was performed on six disease groups. We explored functional differences between disease clusters using curated GO annotation from the BKL. Calculation of preponderance values and GO term assignments were also performed with enrichment P-values reported by the DAVID Functional Annotation Tool [27,28] as a public source of GO annotation. The first three groups used in the analysis match regions of the giant component: the top region mainly comprising cardiometabolic diseases also including parathyroid and pituitary neoplasms, the middle region constituted of leukemia, lymphoma, and immune system-related pathologies, as well as the lower region with solid tissue neoplasms (Fig. 1). In the following, we denote these groups as clusters M, I, and C, respectively. The other three disease groups were obtained from networks shown in Fig. 2, and are in the following denoted as clusters D (Fig. 2A), P (Fig. 2B), and N (Fig. 2C). Enrichment P-values were calculated and compared for complementary sets made up of genes which were specific for each cluster. Respectively, 337 genes, 279 genes, 683 genes, 107 genes, 82 genes, and 130 genes represented cluster M, I, C, D, P, and N. All diseases were associated with at least one gene in corresponding gene sets, except for transient ischemic attack, so that results only apply to 62 of 63 disorders in cluster M.

**Figure 3:**
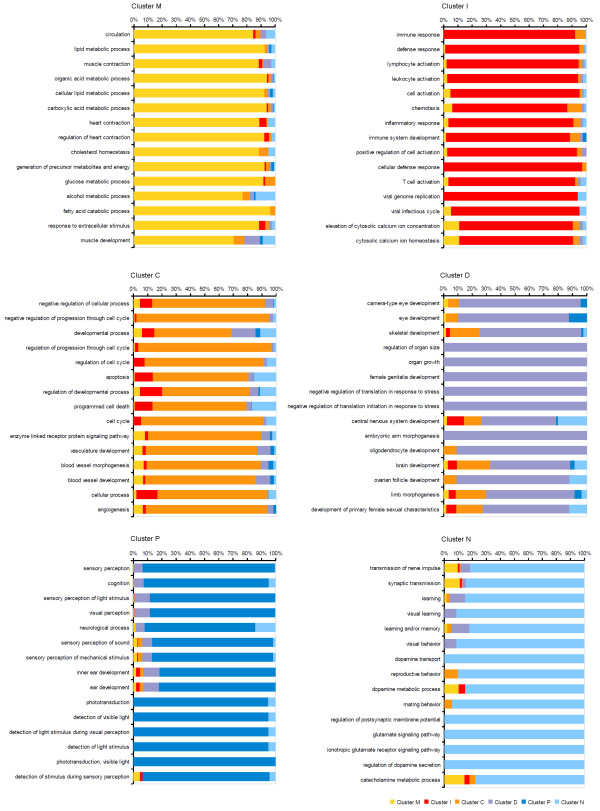
**Preponderant biological functions in disease clusters**. For each of the six disease clusters extracted from the disease map, the figure shows the top 15 GO biological processes ranked by the preponderance value described in the Methods section. This analysis reveals biological functions which are most strongly associated with a certain disease cluster compared to all other clusters. The most preponderant gene classes reflect meaningful features of corresponding disease groups, such as metabolic processes in cluster M, cell cycle and apoptosis functions in cluster C, or genes involved in neurotransmission in cluster N.

Fig. 3 shows the top 15 GO biological processes of each disease cluster ranked by preponderance value. Bar plots indicate the relative importance of a particular biological function in a disease cluster comparing all six enrichment P-values. These results demonstrate preponderance of certain biological functions in each disease cluster. Cluster M was characterized by genes with metabolic and homeostatic functions, whereas immune system-related functions signified cluster I. Furthermore, we found preponderance of apoptotic, angiogenetic, cell cycle and cell developmental functions in cluster C. Cluster D genes showed strongest tendency to encode components of specific developmental and morphogenetic processes. Finally, the analysis revealed preponderance of genes encoding sensorineural processes in cluster P, whereas behavioral and neurological functions were associated with cluster N.

Since the BKL assignments of genes to GO biological processes are manually curated, we carried out the same analysis using enrichment P-values calculated by the DAVID Functional Annotation Tool in order to validate our results with an alternative source of GO annotations. In Additional file 2 we report the top 30 biological processes associated with each disease cluster according to enrichment P-values calculated by the DAVID tool. The topics of categories assigned to disease clusters based on GO annotation of DAVID are in good agreement with those observed in the analysis of curated BKL annotation. A notable difference is the absence of cell cycle categories among the top 30 biological processes assigned to cluster C in the analysis using DAVID. Cell cycle terms were still associated with disease cluster C, albeit with lower ranks than in the BKL analysis. For instance, preponderance values calculated for DAVID enrichment P-values ranked the GO categories "regulation of cell cycle" and "cell cycle" at position 86 and 88 (data not shown), respectively, whereas they were ranked 5^th ^and 9^th ^in the BKL analysis (Fig. 3). Nevertheless, the top ranked biological processes assigned to disease cluster C based on either DAVID or BKL share a common theme of cell proliferation, apoptosis, angiogenesis and developmental pathways. Hence, both resources confirmed that the disease clusters feature biological processes that reflect the type of clustered disorders.

To further explore the relevance of inferred disease associations, we inspected vicinities of some selected disorders defined by a certain similarity level. Here, we made use of the statistical method to extract all diseases associated with a pathology of interest through at least two common causal genes and a similarity P-value below 0.01. In the following, we exemplify three cases of metabolic disorders, namely type 1 diabetes (T1DM), type 2 diabetes (T2DM), obesity, and the neurodegenerative disorder Parkinson disease (PD).

Table 1 shows vicinal diseases obtained at the specified similarity level. While the metabolic pathologies share several vicinal disorders such as coronary disease and insulin resistance, no commonality with PD is found, so that also at a lower significance threshold neurological and metabolic disorders were distinct. Moreover, specific associations with diseases are unveiled. In the obesity network, we find cushing syndrome, cachexia, acromegaly, and polycystic ovary syndrome (PCOS). Cushing syndrome is an endocrine disorder caused by undue exposure to cortisol. Its symptoms include weight gain and obesity. Like acromegaly, cushing syndrome can be caused by pituitary adenoma, which is the case in 70% of cushing syndrome incidences [29]. Clinical associations were previously reported for acromegaly and obesity [30] as well as for obesity and PCOS [31,32]. Cachexia has manifestations that are adverse to obesity, such as loss of weight. As shown in Additional file 3, our analysis suggests ghrelin (GHRL) as a common mechanistic component which was recently discussed in several articles [33,34]. The network connects obesity (yellow node) with vicinal diseases (red nodes, see also Table 1) through common causal genes (blue nodes). Besides GHRL, the set of causal genes shared by obesity and cachexia comprised ADRB2, MC4R, and CNTF (Additional file 3). Furthermore, the T1DM vicinity reflects its immunological etiology, whereas PD is associated exclusively with other neurological disorders (Table 1). Graft vs. Host disease, pancreatitis as well as Sjögren's syndrome are immune system-related disorders which, like T1DM, involve immune response-driven tissue destruction. Connections between PD and schizophrenia as well as attention deficit disorder with hyperactivity are supported by dysregulation of dopamine-dependent neurotransmission shared by these morbidities [35,36], whereas clinical associations of PD with bipolar disorder and with dystonia have been previously described as effects of PD therapy with dopamine agonists, also suggesting common causal mechanisms [37,38].

**Table 1:** Disorders associated with obesity, Parkinson disease, T1DM and T2DM at a P-value threshold of 0.01 and an overlap of at least 2 genes.

Obesity	Parkinson	T1DM	T2DM
Acromegaly	Alzheimer Disease	Coronary Disease	Coronarya Artery Disease

Cachexia	Amyotrophic Lateral Sclerosis	Diabetes Mellitus	Coronary Disease

Cardiomyopathy, Dilated	Attention Deficit Disorder with Hyperactivity	Diabetes Mellitus, Type 2	Diabetes Mellitus

Coronary Disease	Bipolar Disorder	Diabetic Nephropathies	Diabetes Mellitus, Type 1

Cushing Syndrome	Brain Ischemia	Graft vs. Host Disease	Heart Failure

Diabetes Mellitus	Dystonia	Hyperinsulinism	Hypercholesterolemia

Diabetes Mellitus, Type 2	Epilepsy, Temporal Lobe	Insulin Resistance	Hyperinsulinism

Heart Failure	Neurodegenerative Diseases	Pancreatitis	Hypertension

Hyperlipoproteinemia Type II	Parkinsonian Disorders	Sjögren's Syndrome	Insulin Resistance
		
Hypertension	Schizophrenia		Myocardial Infarction
			
Insulin Resistance			Obesity
			
Obesity, Morbid			
			
Polycystic Ovary Syndrome			

In summary, the statistical analysis of causal genes enabled us to find meaningful disease associations. Interestingly, many of these associations correspond to clinical observations of comorbidities and known etiological relationships between diseases as supported by highlighted scientific literature. Examination of disease clusters revealed characteristic biological functions which confirm the causal mechanistic basis of inferred disease relationships. Altogether, our findings suggest that causal molecular mechanisms provide for an expedient principle to gain further insight into the network of human diseases.

### Prediction of causal genes

Having a method to identify meaningful disease associations, our next goal was to apply disease similarities as a starting point for causal gene prediction. Following our previous results, we assumed that gene sets of associated disorders potentially harbor novel mechanistic components of the disease of interest. The short-list of candidate genes was then culled from associated pathologies hypothesizing that frequent co-occurrence in causal gene sets implies functional relationship with a known disease gene (Methods).

In this work, we chose a similarity P-value of 0.01 and a minimal overlap of two causal genes or diseases to infer links between diseases or genes, respectively. The procedure was employed to predict novel disease genes for T1DM, T2DM and obesity, which are listed in Table 2.

**Table 2:** Causal genes predicted for T1DM, T2DM and obesity and supporting literature referenced by PubMed identifiers.

T1DM		T2DM		Obesity	
**Gene**	**PubMed**	**Gene**	**PubMed**	**Gene**	**PubMed**

**AGT**	18413222	**ABCC8**	17259403	**AGTR1**	15878965
			12559865		

**AKT2**		**ADRB1**		**APOA2**	17446329

**APOA4**	16770585	**APOA1**	17654446	**APOA4**	

**CD36**	15737001	**APOA4**	17654446	**APOB**	10706596

**CRP**	15448085	**APOE**	12439646	**CRP**	11390329
					10591334

**CYP19A**1		**CBS**	12198128	**GCG**	19597507

**GCK**		**CD44**	19017033	**GCK**	18483479

**HMGCR**	17941871	**ENPP1**	17143316	**GNAI2**	17928396

**IGF2**		**HGF**	16759302	**HNF1A**	

**ITGA2B**		**IGF1**	15832492	**IGF2**	

**NOS3**	19246226	**IL2**		**ITGA2B**	

**P2RY12**		**IRS2**	9495343	**LCAT**	

**VEGFA**	17513698	**ITGA2B**		**LDLR**	10914685
		
		**LDLR**	12716819	**NR3C2**	18427128
		
		**PAX6**	19130035	**P2RY12**	
			19034419		
		
		**PON1**	10667477	**RYR2**	16793060
		
		**PROC**	17971179	**SSTR5**	12943494
		
		**REN**	15516153	**XDH**	17276354
				
		**RYR2**	15044459		
				
		**SOD1**	18292963		

By manual literature research we could verify the majority of predictions as shown by the PubMed identifiers of relevant research articles given next to corresponding candidate gene symbols. Corroboration of our predictions was least successful for T1DM with 6 of 13 candidate genes left unverified, whereas only 3 of 20 genes proposed to be involved in T2DM, namely ADRB1, IL2, and ITGA2B, were not confirmed.

As an additional step, we performed network analysis of signal transduction molecules encoded by known and predicted causal genes using the network cluster algorithm of ExPlain™ [39]. The algorithm constructs signaling pathways connecting as many molecules from an input set as possible with a distance constraint for reaction cascades. As a result, input molecules are clustered into networks of two or more molecules. These network clusters can be visualized and subjected to other bioinformatic analyses [39]. In our pursuit, the application served two purposes. Firstly, molecular pathways point out potential mechanisms by which known and predicted causal components exert a common function. Secondly, signaling cascades may allude to additional, previously unknown constituents of disease mechanisms. In the following, we examined network clusters of known and predicted causal components of T1DM as well as T2DM.

ExPlain™ reported two network clusters for T1DM. In the following, we provide gene symbols in parentheses where these differ from the protein names reported in ExPlain™ networks. A small cluster consisted of the known causal component CD154 (CD40LG) and the predicted molecule alpha IIb-integrin encoded by ITGA2B (Table 2), providing computational evidence for a role of ITGA2B in T1DM. Additional file 4 shows the larger cluster including ten known causal components (red nodes) and the novel component IGF-2 (IGF2) (green node) connected by other molecules (blue nodes) through activating (black arrows) or inhibitory (red arrows) reactions. By manual literature research, we verified involvement of SOCS3 [40], Jak1 (JAK1) [41], and SHP-1 (PTPN6) [42] in T1DM. Notably, Grb-14 (GRB14) and PTP1B (PTPN1) are known molecular constituents of insulin resistance [43,44] and development of PTP1B inhibitors for therapeutic modulation of insulin sensitivity is an active field of research [45]. While PTP1B and Grb-14 functions were mainly explored with regard to their causal role in T2DM and obesity, the prevalence of insulin resistance in conjunction with type 1 diabetes has recently gained attention [46,47].

We further obtained two network clusters of T2DM molecules shown in Additional files 5 and 6. In a small network (Additional file 5), known causal components ADA and CD26 (DPP4) form a cascade with the predicted causal component CD44 (green node) connected by RANTES (CCL5) (blue node), which harbors promoter polymorphisms associated with type 2 diabetes [48]. The larger network (Additional file 6) comprises 19 known causal components (red nodes) and 5 predicted components, namely activated protein C (PROC), alpha-IIb integrin (ITGA2B), Cu-ZnSOD (SOD1), IRS-2 (IRS2) and IL-2 (IL2) (green nodes). Moreover, scientific literature supports a mechanistic role of several network components, such as PKCdelta (PRKCD) [49], PKCtheta (PRKCQ) [50], GSK3 (GSK3B) [51], p85 (PIK3R1) [52], Rac1 (RAC1) [53], p65PAK (PAK1) [54], Akt (AKT1), PDK1 (PDPK1), and mTOR (MTOR) [55].

Taken together, disease and gene associations successfully predicted causal genes for obesity, T1DM, and T2DM, and scientific literature verified the majority of proposed candidates. Molecular network analysis of T1DM and T2DM gene sets then suggested signal transduction cascades connecting predicted and known causal proteins encoded by respective genes. Additional constituents of causal disease mechanisms were inferred along with molecular pathways and a good part of them (more than 1/3) were supported by literature evidence. Notably, many of the cited research articles investigated respective causal components as therapeutic targets for T1DM or T2DM. These results demonstrate the utility of causal mechanism-based disease analysis for inference of novel disease genes.

### Evaluation of causal gene predictions

**Figure 4:**
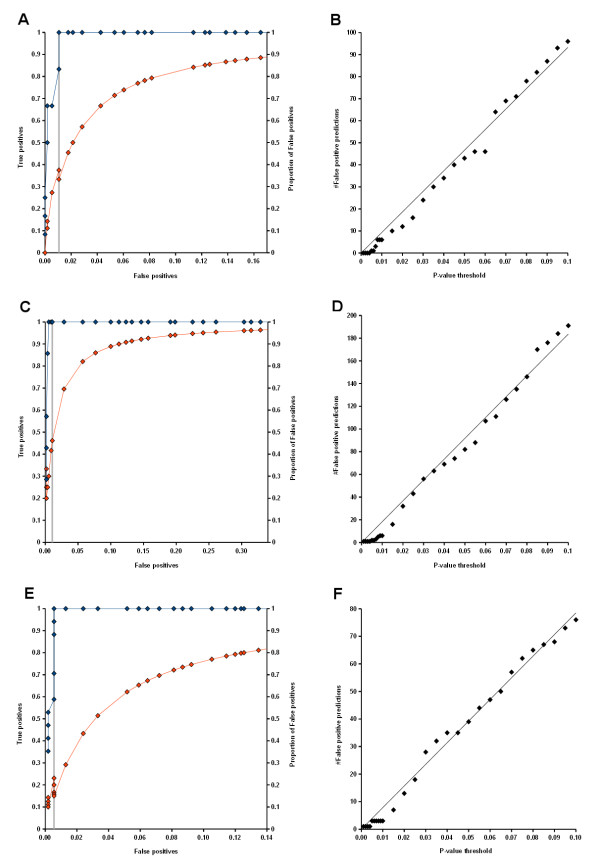
**Characteristics of the method for disease gene prediction**. The left side shows ROC curves and plots of the proportion of false positives among predicted genes for obesity (A), T1DM (C), and T2DM (E). Numbers of predicted false positive genes obtained at P-value thresholds from 0.001 to 0.1 are shown on the right side (B - obesity, D - T1DM, F - T2DM).

We used literature-verified causal genes that were predicted for obesity, T1DM, and T2DM (Table 2) as reference sets to calculate false positive and true positive rates at different P-value thresholds in the range from 0.001 to 0.1. As before, the same P-value cut-off was imposed on disease similarity as well as gene similarity and a minimal overlap of two genes or diseases was required. Fig. (4A, C, and 4E) shows receiver operator characteristics (ROC) (red curves) and plots of the proportion of false positive predictions among all predicted disease genes (blue curves). True positive rates were estimated from literature-verified causal gene sets, which comprised 12, 7, and 17 genes for obesity, T1DM, and T2DM, respectively (Table 2). False positive rates were based on all other, non-verified genes. In the ROC plots, the location of P-value cut-off 0.01, that was originally applied to obtain reference sets is indicated by gray lines. ROC curves are supported by plots of the proportion of false positives among all predicted disease genes at a given threshold. Both quantities show that the true positive rate grows faster than the false positive rate with increasing P-value threshold. The ROC curves present an idealized trend, since false positive rates were calculated from a much larger number of genes than true positive rates. Yet, in all three examples the proportion of false positive genes decreased with more stringent thresholds both above and below the reference cut-off. According to this analysis, the best precision values ranged from 80% to 90%. We defined the best precision threshold as the P-value cut-off that achieved prediction of a maximal proportion of true positives while requiring at least one false positive gene. Optimal precision values were 86% (6 true positive genes), 80% (4 true positive genes), and 90% (9 true positive genes) for obesity, T1DM, and T2DM, respectively. Hence, disease similarities and causal gene comparison discriminated against false positive genes and selected for true disease genes.

We examined how the number of predictions correlated with P-value thresholds and observed an approximately linear dependence in all three examples (Fig. 4B, D, and 4F). This shows that the P-values effectively controlled the number of predictions, albeit the rates of change were not the same for the three disorders.

Cross-validation (CV) was performed to evaluate the robustness of our results with perturbed disease/gene association data. We carried out 20 rounds of cross-validation for obesity, T1DM, and T2DM by removing 5 randomly chosen genes from their causal gene sets. Poisson parameters were re-estimated for diseases and for genes based on 10^5 ^random gene sets and 10^5 ^random disease sets, respectively, using the modified association data. Subsequently, we predicted causal genes for each of the disorders at a P-value threshold of 0.01 and an overlap threshold of 2.

**Figure 5:**
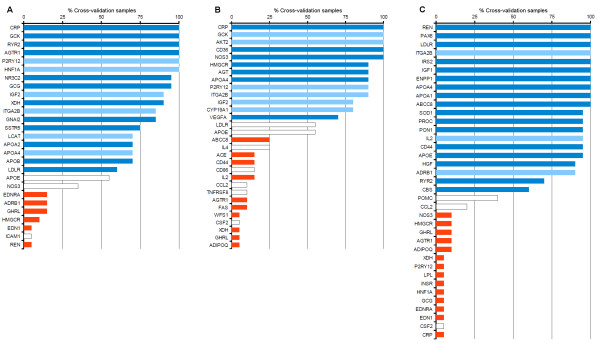
**Cross-validation of disease gene predictions**. Each bar plot shows all genes predicted during cross-validation for obesity (A), T1DM (B), and T2DM (C). Blue bars indicate genes that were predicted with the unperturbed set of disease/gene, some of which could be verified with literature evidence (dark blue). Red bars correspond to known disease genes omitted from cross-validation samples, whereas white bars signify genes newly predicted in the validation.

The bar plots in Fig. 5 list all genes that were predicted in the course of 20 cross-validation rounds and show the fraction of samples in which the genes were encountered. For all three disorders, the genes that were predicted in the unmodified data set and were reported in Table 2 (blue bars) also appeared in more than 50% of the cross-validation samples, and the majority of these genes was encountered more than 75% of the time. While several genes were newly predicted during cross-validation (white bars), none of them was predicted as often as those from the original set. Furthermore, most of the newly predicted genes were known disease genes that had been removed from the causal gene sets (red bars). Some new genes were incurred in a considerable amount of samples such as APOE and NOS3 in obesity (Fig. 5A), LDLR, APOE, and IL4 in T1DM (Fig. 5B), as well as POMC and CCL2 in T2DM (Fig. 5C). We think, that this variability can at least in part be explained by the sampling of random disease or gene sets in the empirical estimation of Poisson parameters. For instance, in the T2DM analysis CCL2 was predicted in four cross-validation rounds due to similarity with AGTR1 at P-values ranging from 8.77e-3 to 9.998e-3. Likewise, POMC was similar to the T2DM gene SERPINE1 with P-values from 9.62e-3 to 9.97e-3 in 8 CV rounds. The proximity of these P-values to the cut-off suggests the sampling process as the source of that variability, because small changes in the regression model could shift the estimated similarity P-value above or below the threshold. Nevertheless, the method robustly selected certain genes most of the time, whereas there is a visible distinction to other, non-disease genes that were detected infrequently (Fig. 5).

**Figure 6:**
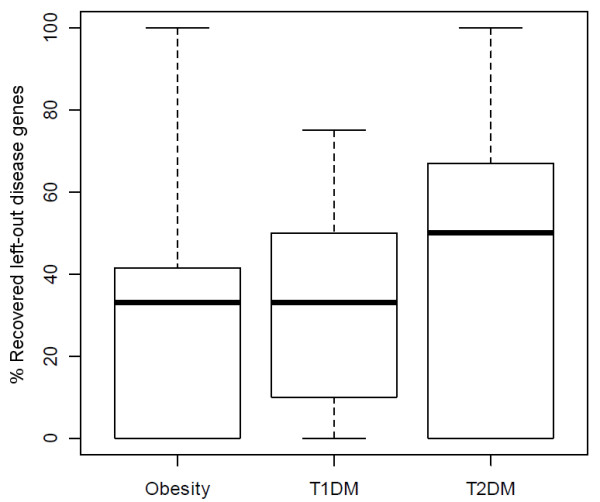
**Box plots of recovery rates estimated by cross-validation**. The box plots of distributions of the fraction of known disease genes that were omitted from causal gene sets of obesity, T1DM and T2DM and recovered by disease gene prediction.

Furthermore, we examined the recovery of left-out disease genes and illustrate the obtained statistics in box plots (Fig. 6). The method achieved recovery rates between 30% and 60%, yet with large deviations. Hence, the procedure could only identify a minor part of left-out genes. We speculate, that this performance is due to the sparseness of the underlying data and the recovery rates reflect the proportion of known disease genes that could be identified given the imposed thresholds.

In summary, cross-validation demonstrated that the method robustly produced a limited set of genes which preferably included the originally reported gene predictions. We could observe effects of sampling random disease or gene sets in the empirical estimation of Poisson parameters. Increasing the number of random sets would mitigate the variability inherent to the sampling procedure. This does not represent a major drawback, since the regression analysis needs to be conducted only once and parameter estimates can then be used in subsequent comparisons. Furthermore, we assume that recovery of known disease genes was limited to certain fraction of the causal gene sets due to the low coverage of underlying disease/gene associations. This suggests that other types of information such as molecular interactions could complement the predictions entirely based on disease/gene associations.

We therefore tested the utility of combining predictions derived from disease/gene associations with a method that employs molecular interactions. The GeneWanderer is a tool that applies a global network distance measure to rank candidate genes according to their context to known disease genes in a network [56]. The algorithm assigns a distance value to candidate genes based on a random walk with restart (RWR), which reflects how well candidate genes are connected to modules of disease genes [56]. RWR distance values are higher for genes that are well connected to known disease genes and were successfully applied to prioritize candidate genes [56]. We compared the ranks of distance values obtained with and without inclusion of genes predicted on the basis of disease/gene associations for known disease genes that were omitted from the cross-validation samples. Thus, for each of the left-out disease genes, we calculated RWR distance values using either only the truncated set of disease genes or predicted genes in addition. In the following, we denote RWR distance values as network score. An interaction network containing 10486 genes and 109089 interactions was compiled from the IntAct [57], BioGRID [58], and Reactome [17] databases (Additional file 7). We used the ranks of network scores of the test disease genes among all genes of the network to compare the performance with and without addition of genes predicted by disease/gene associations in each CV sample.

**Figure 7:**
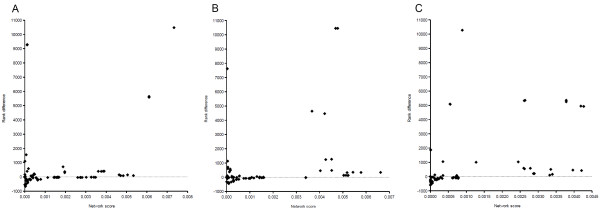
**Network scores and rank differences for tested disease genes**. Network scores and associated rank differences compared for reduced disease gene sets only and with inclusion of genes predicted on the basis of disease/gene associations. Dots represent known disease gene omitted from cross validation set generated for obesity (A), T1DM (B), and T2DM (C). See text for further description.

An improved rank of the network score would enhance the possibility to correctly prioritize a disease gene, because it would increase its chance to be highly ranked among candidate genes. Ranks were calculated as 10486-r, where r is the conventional rank order, so that higher values correspond to better network scores. Fig. 7 shows the results of this analysis for obesity (Fig. 7A), T1DM (Fig. 7B), and T2DM (Fig. 7C). Dots represent disease genes that were omitted from CV sets and are located according to the highest of the network scores calculated with or without inclusion of predicted genes (x-axis) and the corresponding rank difference (y-axis). Rank differences for test disease genes are positive if the addition of predicted genes to the attenuated disease gene set improved the rank of the network score. The plots show that the genes predicted by disease/gene associations enhanced the network scores of many test genes. The cases where the ranks decreased (values below zero on the y-axis) mostly involved genes with a low network score, which were thus not well connected to other disease genes in the interaction network. In addition, the decrease was never lower than -1000. On the contrary, inclusion of predicted genes could substantially improve the network scores for several genes. Importantly, higher network scores showed a clear tendency for improvement. For instance, in the T2DM comparison, ranks of network scores greater than 0.001 were always better than with the attenuated disease gene set alone (Fig. 7C). Since higher network scores signify a smaller global distance to the set of input disease genes, this demonstrates that addition of genes that were predicted on the basis of disease/gene associations supported the network scores of test genes in the vicinity of modules of disease genes. These results show that disease gene prediction can benefit from combining disease associations with analysis of molecular interactions. Often only a handful of causal genes are known for a disorder. Our analysis suggests that consideration of genes from diseases that are likely to involve similar molecular mechanisms can enhance the prediction of novel disease genes.

## Discussion

Knowledge about components of causal mechanisms has proven useful to analyze relationships between human diseases. The definition of causal genes applied in the BKL includes genotype associations, yet also covers other sources of evidence for involvement in causal molecular systems. This may be of importance taking into account that the activity of gene products in the context of molecular networks may bias the ability of genes to harbor pathological mutations as shown by several studies [59-61]. Probably, more or less complex patterns of genetic variation contribute to every disease. However, their functional effects become manifest in molecular interactions, where networks of proteins, yet also protein/DNA interactions, take an important part and genetic alterations are one of many possibilities to induce derangement [62].

On the foundation of causal disease/gene associations, we built a method to quantify the similarity of two diseases that accounts for unequal frequencies of genes in the entire set of associations. To provide a familiar and intuitive quantity, the presented method reports a P-value for the overlap of causal gene sets. At standard P-value thresholds, 0.001 and 0.01, the statistical analysis revealed meaningful disease associations as demonstrated by constructing a map of human diseases, GO analysis of disease clusters and inspection of vicinal disorders for obesity, T1DM, T2DM and PD at the lower threshold.

Human disease networks were previously studied with respect to physiological disease classes [10,14]. Here, we first constructed the disease map and afterwards demonstrated that biological processes coincided with well-known attributes of clustered disorders as confirmed by manually curated GO biological process annotation of the BKL as well as GO annotations available through the DAVID Functional Annotation Tool. Furthermore, we observed that the giant component of our disease map as well as the vicinities of obesity, T1DM, and T2DM clustered components of the cardiometabolic syndrome. Notably, the disease vicinities that were explored in more detail reflected not only similarities between obesity, T1DM, and T2DM, but captured also specific relationships such as connections to immunological disorders in the T1DM vicinity. When we applied causal disease associations to predict new disease genes, the majority of predictions proposed for obesity, T1DM, and T2DM could be supported by references to scientific literature. Altogether, our results corroborate that the inferred disease associations reflect common molecular mechanisms and indicate applications for disease gene prediction as well as disease classification and definition.

Limitations of current standard classifications were previously challenged with regard to molecular or complex systems approaches [63,64]. Protein-protein interactions and molecular pathways have been employed to identify disease relationships [13,14]. So far our method for disease comparison left molecular interactions unspecified and addressed only the overlap of causal gene sets. A first step towards incorporating molecular networks into our analysis was taken by clustering causal components in signal transduction networks. As evidenced by scientific literature, the network clusters contained many known causal components and therapeutic targets. Furthermore, we translated the analysis of disease associations and of causal gene associations into a method to select for new disease genes. While disease gene prediction entirely based on experimentally verified disease/gene associations faced limitations originating from the sparseness of the available data, we could show that a combination of disease associations and molecular network analysis enhanced the possibility to identify new disease genes. Incorporation of molecular interactions is therefore an important area for further development, where greater fidelity with molecular systems that underlie disease mechanisms can be achieved.

We would like to point out that our method for prediction of causal genes relied on four cut-off values consisting of a P-value and a minimal overlap parameter in the first and the second step (Fig. 9). It may be difficult to tune each of the parameters to achieve optimal results. Throughout this work, we set identical cut-offs for disease and gene similarity and confined analyses to standard P-value thresholds (0.001 or 0.01). Furthermore, the overlap threshold was always set to a small value of 2 with the purpose of controlling a minimal level of shared causal genes or diseases. We think that with this setting the overlap cut-off sufficiently complements the P-value. As demonstrated, the number of false positives grows linearly with the P-value threshold (Fig. 4), so that this parameter lends itself to further adjust the algorithm. One possibility is to choose a number of predictions admissible for validation and to select a P-value cut-off that satisfies this constraint. For this type of approach our method offers, in addition to using a common value for disease and gene similarity, the possibility to fix the disease similarity threshold (e.g. to 0.01) and to subsequently rank predicted causal genes according to overlap P-values with known disease genes. Furthermore, the method achieved best precision for obesity, T1DM, and T2DM at P-value thresholds of 0.005, 0.003, and 0.01, respectively. At a P-value of 0.005 the observed precision for T1DM and T2DM was still at least 75%. This indicates that the range from 0.001 to 0.01 is suitable to choose a threshold for causal gene prediction and suggests 0.005 as a possible starting point.

In our efforts to verify the inferred disease associations, we were able to highlight many instances of known clinical associations and comorbidities, suggesting that these are special cases of related pathologies sharing causal mechanisms which also connect them etiologically. Interestingly, these findings inversely confirm a previous study, where co-occurrence of disorders in medical records was used to predict genetic overlap [9]. Validation of disease co-prevalences often requires laborious population studies. According to the results of this work, the decision to mount a study could be supported by testing hypotheses about disease associations computationally. Simultaneously, shared causal components provide insight into the molecular basis of etiological disease relationships and suggest potential diagnostic markers.

So far, different methods have been proposed to investigate human disease associations. A main difference lies in the representation of disease entities by features that are eventually compared to obtain a figure of similarity. While this work focused on genes that were manually classified as causal disease genes, other approaches used clinical characteristics, phenotypes, or genes and pathways [5,8,14]. Each choice of feature representation involves advantages and disadvantages with respect to quality, coverage, or detail of information. For instance, manual curation promises greater quality than computationally derived annotation, but its coverage is often inferior. Furthermore, associated genes capture disease components in finer detail than descriptions of clinical characteristics, but we assume that for a disease the latter are more often defined than associated genes. It is therefore of importance to compare the different approaches to recognize and validate strengths and weaknesses.

To the best of our knowledge, no reference set has been established to systematically examine the ability of different methods to correctly identify disease associations, so that a necessary step towards such a comparison is to assemble a set of known disease links.

Another future direction will be to combine different levels of information such as causal genes, affected biological processes, and clinical characteristics to gain further insight into disease subtypes and corresponding mechanisms. Of interest are phenotypically similar disease subtypes that present different molecular mechanisms as well as similarities on the molecular level that cannot be mapped to known clinical characteristics. Identification of such disease subtypes and of "hidden" disease similarities may open new avenues to develop therapeutic approaches for respective disorders.

## Conclusions

We developed a novel approach to analyze human disease associations and demonstrated its utility in several application areas. Causal molecular mechanisms present a unifying principle for disease classification and definition, analysis of clinical disorder associations, as well as prediction of disease genes, therapeutic targets and diagnostic markers. According to the definition of causal disease genes applied in this study, these results are not restricted to genetic disease/gene relationships. This may be particularly useful for the study of long-term or chronic illnesses, where pathological derangement due to environmental or as part of sequel conditions is of importance and may not be fully explained by genetic background. The possibility to identify common molecular mechanisms for clinically associated disorders enables further insight into disease interactions. First steps in that direction were presented in this work for obesity and diabetic disorders, as constituents of the cardiometabolic syndrome. An important conclusion from this work is that components of molecular mechanisms characterize associated diseases. Using this knowledge enables identification of disease associations, which reflect common molecular mechanisms, and provides for a starting point to identify missing causal components. Making use of such disease associations and consideration of knowledge about molecular interactions can be combined to handle limitations imposed by the sparseness of experimentally verified, curated disease/gene associations. Future lines of research will include incorporation of molecular interactions into the method for disease comparison and development of software tools that exploit the findings of this work.

## Methods

### Causal disease/gene associations

Manually collected information on causal disease/gene associations was obtained from the BIOBASE Knowledge Library™ (BKL) [19]. The BKL groups disease/gene associations into four types, *correlative*, *causal, preventative*, and *negative*, depending on the conclusion that can be drawn from a relevant research article. In this study, we used only associations of the causal and of the preventative type. Causal relationships are derived from experiments, which confirm or suggest the hypothesis that a gene encodes a product whose deranged activity entails a disease or a certain condition as part of a disease. The derangement may be inheritable or emerge during disease onset or progression. Preventative disease/gene associations additionally evince that experimental evidence is available for a therapeutic effect of modulating the deranged activity. In this article, we denote the respective genes as causal genes. To ensure a certain level of annotation for disease comparison, we considered only disease entities with at least five causal genes. The eventual data set comprised 375 diseases and 3051 causal genes connected by a total of 9871 disease/gene associations. Data used in this study are available upon request.

The BKL specifies diseases using MeSH descriptors [4], which constitute a hierarchy of broader and narrower subject headings. The hierarchical structure of MeSH descriptors allows for incorporation of disease-related scientific information into the knowledgebase, even when the disorder to which an article pertains is not distinguishable at the most specific level. Inference of disease associations performed in this work ignored hierarchical dependencies. Hence, it was anticipated that similarities between some disorders merely reflect the underlying MeSH structure.

### Disease comparison

**Figure 8:**
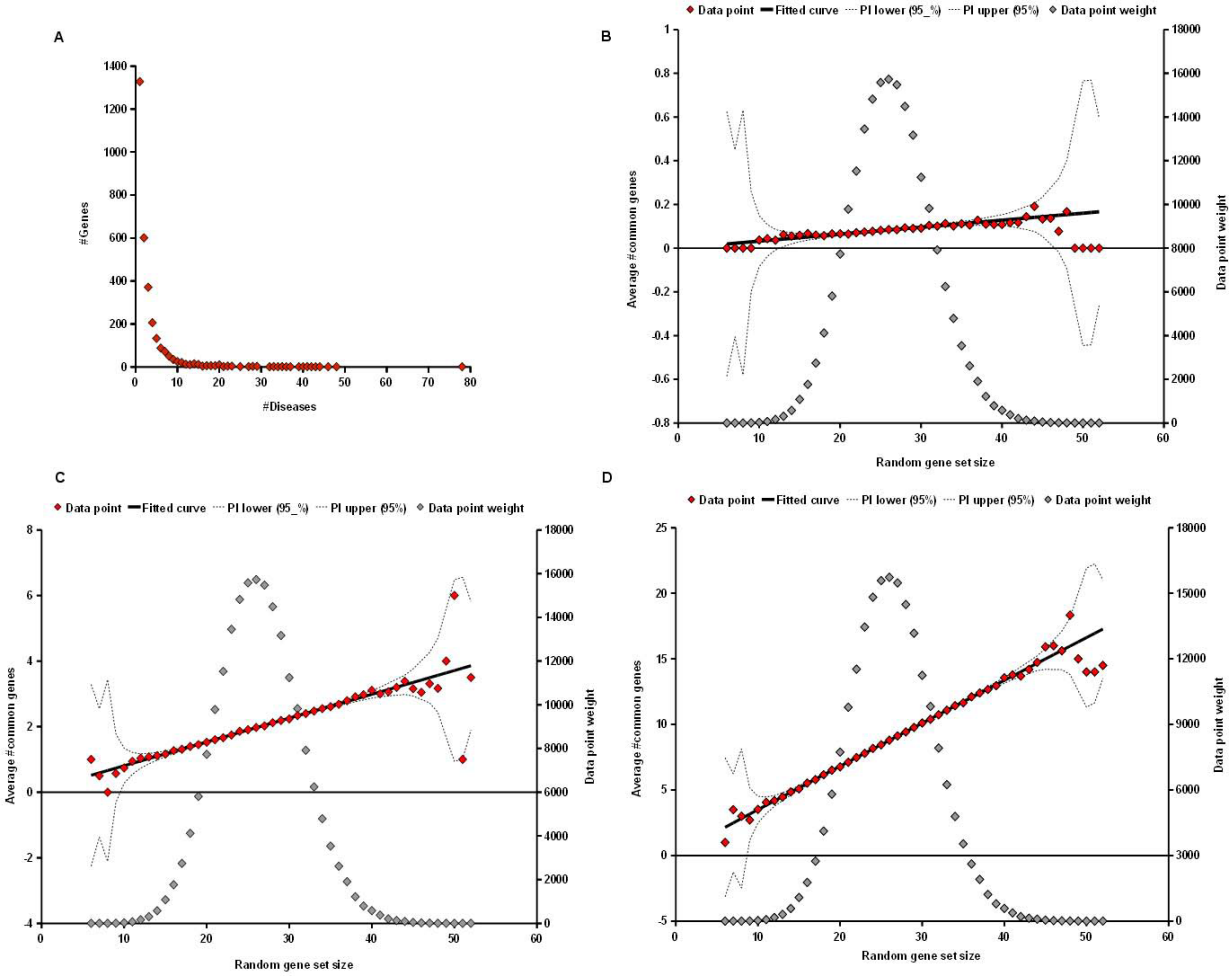
**Distribution of disease/gene associations and regression analyses of gene overlap as a function of random gene set size**. (A) Distribution of disease/gene associations. The plot shows how many genes (y-axis) are linked to a certain number of diseases (x-axis). The majority of genes (about 86%) in the BKL data set are not associated with more than 5 disorders, whereas other genes are much more strongly connected. These differences in disease links per gene were taken into account by generating random sets containing genes with the same frequency as the original data. (B-C) Regression functions obtained for calcinosis (B), T2DM (C), and prostatic neoplasms (D). Given a causal gene set, the mean overlap with a random gene set is a linear function of the random gene set size. Deviations from regression curves are observed for small and large random gene sets, which are supported by fewer samples as shown by corresponding data point weights and predictive intervals.

We estimated the statistical significance of the number of common causal genes to quantify similarity between two diseases. The one-tailed Fisher test, which is often used to evaluate enrichment of GO categories, could be applied to identify high numbers of shared genes. However, the Fisher test assigns the same sampling probability to all genes. As shown in Fig. 8A, this assumption does not hold for the BKL data set. Most genes (about 86%) were connected to not more than five diseases, whereas other genes had much higher numbers of associations, up to the extreme of 78 links (about 21% of the 375 diseases) to tumor necrosis factor (TNF). Consequently, disorders involving TNF are more likely to overlap than other diseases whose causal genes may have only few associations. We therefore developed a procedure that takes this property of the data set into account. To this end, a sampling approach was implemented to generate random gene sets in which causal genes occur with the same frequency as in the original data.

It turned out that for a given disease, the mean overlap with random gene sets follows a linear function of the random gene set size, as shown in Fig. 8 for calcinosis (Fig. 8B), T2DM (Fig. 8C), and prostatic neoplasms (Fig. 8D) with 5, 89, and 393 causal genes, respectively. To estimate the statistical significance of an observed number of shared genes, we therefore predicted the mean overlap using a regression model and used the conditional mean estimate to calculate a P-value assuming a Poisson law. A large number (200 K) of random gene sets was generated by sampling one out of all 375 diseases with probability 1/375 for each of the 3051 causal genes and assigning the gene to the respective gene set if the sampled disease was associated with that gene. Each causal gene set, representing a disease of the original data, was compared to the collection of random gene sets to subsequently obtain a model for the dependence of the mean number of common genes on the size of a random gene set by weighted linear regression. The latter was conducted as follows. Let *A *and *R *denote the causal gene sets of a disease from the original data and a random gene set, respectively. The data for regression analysis consisted of points *P*(*X ;Y*) and corresponding weights *W(X) *as defined in (1-3), where |X| denotes the cardinality of a set.

(1) X=|R|

(2) $$Y=\frac{\sum_{\left|R\right|=X}\left|A\cap R\right|}{\#\left\{R:\left|R\right|=X\right\}}$$

(3) W(X)=#{R:|R|=X}

We implemented a step-wise procedure to select a suitable regression model. The procedure examined all linear models specified by equation (4), where $\hat{\lambda }$ is the estimated mean number of common genes, *n *is the number of genes in a random gene set, and $\hat{\beta }$ represents a parameter of the regression function. Models with or without intercept ${\hat{\beta }}_{0}$ were treated explicitly by the extra coefficient *c*.

(4) $$\hat{\lambda }=c\cdot {\hat{\beta }}_{0}+\sum_{k=1}^{k_{max}}{\hat{\beta }}_{k}\cdot n^{\frac{1}{k}};c\in \left(0,1\right);k_{max}\in \left\{1..4\right\};$$

Iterating over all eight combinations of *c *and *k*_max_, the model with lowest Akaike information criterion (AIC) value [65] was chosen among all models whose function was both non-decreasing and predicted only positive mean responses over the whole range of gene set sizes of the original data. Eventually, we used regression estimates to calculate the statistical significance for a number of shared genes assuming a Poisson distribution for gene overlap counts using equation (5), where *x *denotes a number of genes shared by two diseases and $\hat{\lambda }$ represents the Poisson distribution parameter obtained from the regression model.

(5) $$P\left(X\geq x\right)=1-e^{-\hat{\lambda }}\sum_{0\leq k<x}\frac{\hat{\lambda }}{k!}$$

As Poisson parameter estimation was performed for each of 375 diseases, pairwise disease comparison ensues two P-values. We therefore summarized P-values by calculating their geometric mean as defined in (6) in order to obtain a single quantity for each disease pair. In equation (6), *A *and *B *denote gene sets of diseases from BKL and *P*_A _and *P*_B _represent P-values calculated with respective models.

(6) $$P\left(X\geq x\right)=\exp(\frac{1}{2}\cdot \left(\log\left(P_{A}(X\geq x\right)\right)+\log\left(P_{B}\left(X\geq x)\right))\right);x=\left|A\cap B\right|;\text{​}\text{​}\text{​}\text{​}\text{​}$$

The functions lm and summary.lm of the R statistical computing environment [66] were used to estimate regression models and to calculate AIC values.

In the course of disease gene prediction, the described procedure was adopted to estimate P-values for the number of diseases shared by causal genes.

### Statistical analysis of biological processes

Functional characteristics of disease clusters were analyzed with Gene Ontology Biological Processes [15]. Among the three GO vocabularies for biological processes, cellular components, and molecular functions we selected the biological process ontology, because its terms were deemed to best represent molecular mechanisms that may be targets of derangement in disease. Biological process terms describe biological objectives accomplished via one or more ordered assemblies of molecular functions. In the majority of cases, more than one gene contributes to a biological process, whereas GO molecular functions denote biochemical activities of individual genes [15]. Several biological processes are well known targets of disease mechanisms such as cell cycle (GO:0007049) in cancer or immune response (GO:0006955) in auto-immune or infectious diseases.

In order to compare the importance of certain GO biological processes among disease groups, we first calculated one-tailed Fisher test P-values to quantify enrichment of GO categories in respective gene sets and assigned a category to the disease group with lowest Fisher test P-value. Biological functions more strongly associated with one disease group compared to others were found by calculating preponderance values according to equation (7). The preponderance values were used to rank categories allocated with a certain disease group. In (7), the P_i _are Fisher test P-values of *N *disease groups arranged in non-decreasing order. The preponderance value *Prep *is the smallest difference of P-values weighted by the relative proportion of the most significant P-value after logarithmic transformation.

(7) $$Prep\left(GO\right)=\Delta \left(\log_{10}\left(P_{1}\right),\log_{10}\left(P_{2}\right)\right)\cdot \frac{\log_{10}\left(P_{1}\right)}{\sum_{i}\log_{10}\left(P_{i}\right)}$$

with

$$i=1\;\;\;..\;\;N;P_{1}\leq P_{2}\leq ..\leq P_{N};P_{1}<1$$

This analysis was carried out with manually curated GO biological process annotations from the BKL [19] as well as with GO annotations available through the DAVID Functional Annotation Tool [27,28].

### Prediction of causal disease genes

**Figure 9:**
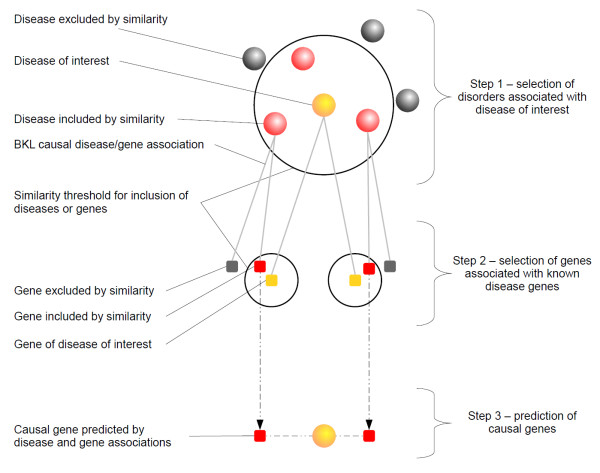
**Workflow for causal gene prediction**. The schema illustrates the workflow to predict causal genes for a disease of interest. In step 1, similar diseases were gathered on the basis of the disease similarity threshold (similarity P-value and overlap threshold). For example, if obesity were the disease of interest and a P-value of 0.01 as well as a minimal overlap of two causal genes were applied as similarity cut-off, this step would select the vicinal disorders of obesity described in Table 1. Step 2 only considered genes of similar diseases which satisfied the similarity threshold with known causal genes of the disease of interest. These genes were eventually predicted as causal genes of the disease of interest (Step 3).

The approach to analyze overlap statistics was applied to construct a method for prediction of causal genes for a disease of interest, which is described in Fig. 9. In the first step, we selected diseases that were similar to the disease of interest at a certain threshold. The selected diseases were hypothesized to potentially share molecular mechanisms with the disease of interest, so that their associated genes could present novel candidate genes. In the second step, we tested for similarity between genes associated with the disease of interest and genes of selected disorders. Here, we assumed that causal genes that overlap in a significant number of disorders may function in a common mechanism. Genes that were associated with a disease selected in the first step and shared a sufficient number of disorders with a known disease gene were then predicted as causal genes for the disease of interest. In this work, gene comparisons were carried for genes with at least 5 disease associations yielding a set of 706 genes linked to a total of 1060 diseases.

### Molecular network clusters

Signal transduction networks of molecules encoded by known and predicted causal genes were constructed by the network cluster tool of ExPlain™ [36]. The algorithm searches for shortest paths of maximally three reaction steps between input molecules. Information about signaling reactions is taken from the TRANSPATH™ database [67]. The ExPlain tool identifies networks, so-called network clusters, which connect a maximal number of input molecules given the distance constraint.

### Network layout and visualization

Layout and visualization of disease networks as well as disease/gene networks were performed with the yED graph editor developed by yWorks [68].

## Authors' contributions

PS conceived of the study, carried out the work and wrote the manuscript, MK provided BKL disease/gene associations, NV developed the network cluster algorithm. AK and EW provided advice on the work. All read and approved the final manuscript.

## Supplementary Material

Additional file 1 **Supplement 1**. Table of disease groups comprising 2 - 6 disorders. Numbering takes into account other disease groups shown in Fig. 1 and 2. Click here for file

Additional file 2 **Supplement 2**. Comparison of GO biological processes among six disease clusters. Click here for file

Additional file 3 **Supplement 3**. Disease network obtained for obesity and common causal genes. Click here for file

Additional file 4 **Supplement 4**. Network of causal components obtained for T1DM. Click here for file

Additional file 5 **Supplement 5**. Small network of causal components obtained for T2DM. Click here for file

Additional file 6 **Supplement 6**. Large network of causal components obtained for T2DM. Click here for file

Additional file 7 **Supplement 7**. Interaction network compiled from IntAct, BioGRID, and Reactome databases. Click here for file
