# Supplementary material for: Molecular mechanistic associations of human diseases
Source: BMC Syst Biol. 2010 Sep 6;4:124. doi: 10.1186/1752-0509-4-124 (PMC2946303; doi:10.1186/1752-0509-4-124)
Supplement: Additional file 1 — Supplement 1. Table of disease groups comprising 2 - 6 disorders. Numbering takes into account other disease groups shown in Fig. 1 and 2. [file 1752-0509-4-124-S1.DOC]

Disease groups not illustrated in Figures 1 and 2

| **Six diseases** | |
| --- | --- |
| **5** | Hyperpituitarism; Pituitary Diseases; Hypothyroidism; Graves Disease; Thyroiditis, Autoimmune; Autoimmune Diseases |
| **Four diseases** | |
| **6** | Puberty, Precocious; Hypogonadism; Endometriosis; Polycystic Ovary Syndrome |
| **7** | Hepatitis C, Chronic; Hepatitis C; Hepatitis B; Hepatitis B, Chronic |
| **8** | Acrocephalosyndactylia; Craniosynostoses; Urinary Bladder Neoplasms; Carcinoma, Transitional Cell |
| **Three diseases** | |
| **9** | Periodontitis; Giant Cell Tumor of Bone; Osteitis Deformans |
| **10** | Hair Diseases; Xeroderma Pigmentosum; Cockayne Syndrome |
| **11** | Kidney Failure; Kidney Diseases; Liver Diseases |
| **12** | Immunologic Deficiency Syndromes; Severe Combined Immunodeficiency; Hypergammaglobulinemia |
| **13** | Ichthyosis; Keratoderma, Palmoplantar; Hyperkeratosis, Epidermolytic |
| **14** | Ataxia; Cerebellar Ataxia; Spinocerebellar Ataxia |
| **15** | Pheochromocytoma; Adrenal Gland Neoplasms; Adrenal Hyperplasia, Congenital |
| **16** | Cystic Fibrosis; Crohn Disease; Colitis, Ulcerative |
| **17** | Limb Deformitis, Congenital; Polydactyly; Syndactyly |
| **18** | Graft Rejection; Hemoglobinuria, Paroxysmal; Malaria, Falciparum |
| **19** | Carcinoma, Papillary; Thyroid Neoplasms; Carcinoma, Pancreatic Ductal |
| **20** | Zellweger Syndrome; Peroxisomal Disorders; Adrenoleukodystrophy |
| **Two diseases** | |
| **21** | Osteoporosis; Osteoporosis, Postmenopausal |
| **22** | Respiratory Distress Syndrome, Adult; Neutropenia |
| **23** | Myasthenic Syndromes, Congenital; Myasthenia Gravis |
| **24** | Leukemia, Monocytic, Acute; Leukemia, Myelomonocytic, Acute |
| **25** | Kidney Failure, Chronic; Kidney Diseases, Cystic |
| **26** | Ínfertility, Male; Oligospermia |
| **27** | Hodgkin Disease; Lymphoma, Large-Cell, Anaplastic |
| **28** | Hypersensitivity, Immediate; Chlamydia Infections |
| **29** | Hereditary Motor and Sensory Neuropathies; Charcot-Marie-Tooth Disease |
| **30** | Skin Diseases; Ectodermal Dysplasia |
| **31** | Growth Disorders; Dwarfism |
| **32** | Leiomyoma; Uterine Neoplasms |
| **33** | Cleft Lip; Cleft Palate |
