# Supplementary figures and images for: Molecular mechanistic associations of human diseases
Source: BMC Syst Biol. 2010 Sep 6;4:124. doi: 10.1186/1752-0509-4-124 (PMC2946303; doi:10.1186/1752-0509-4-124)

Cluster M

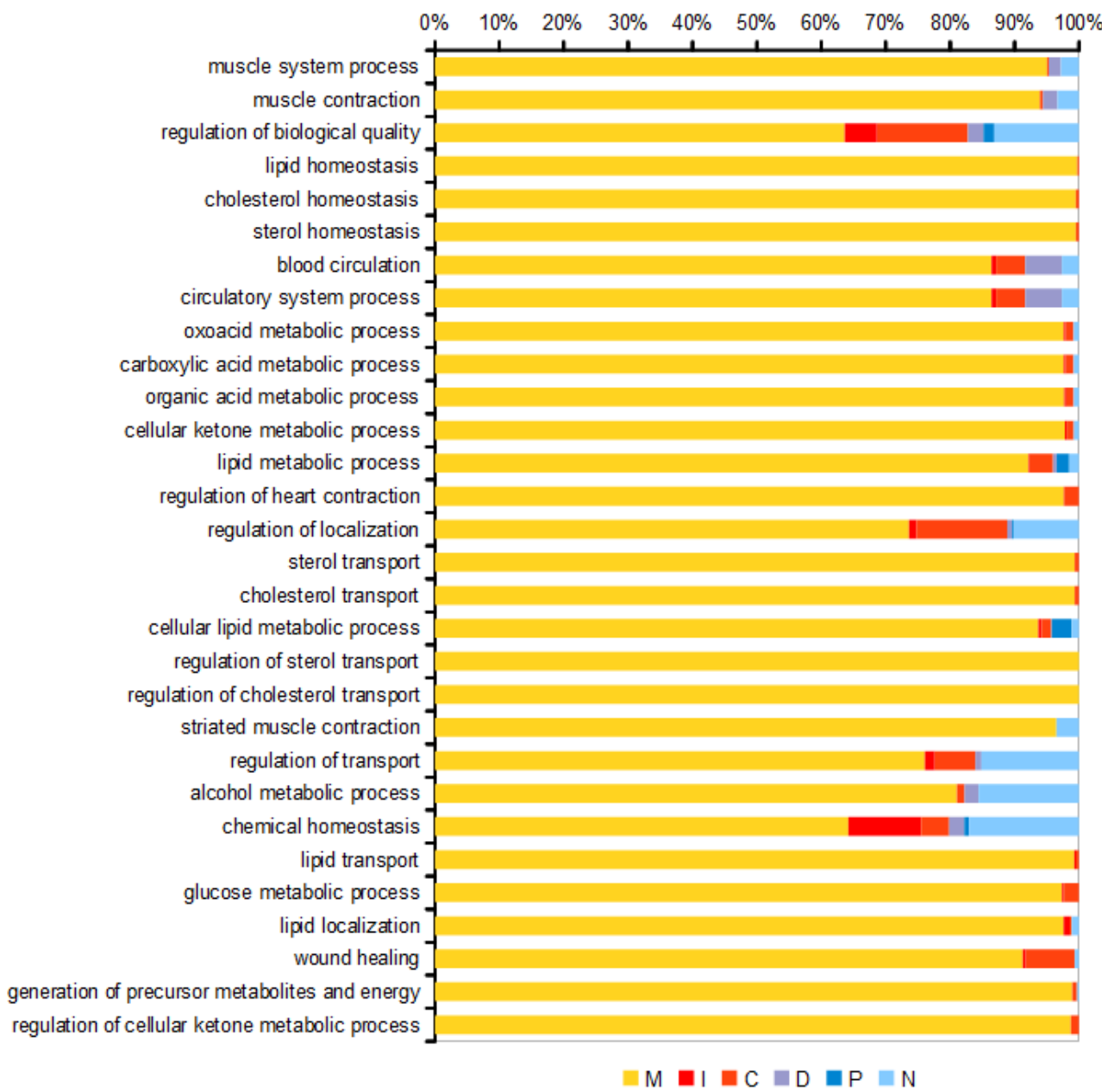

Cluster I

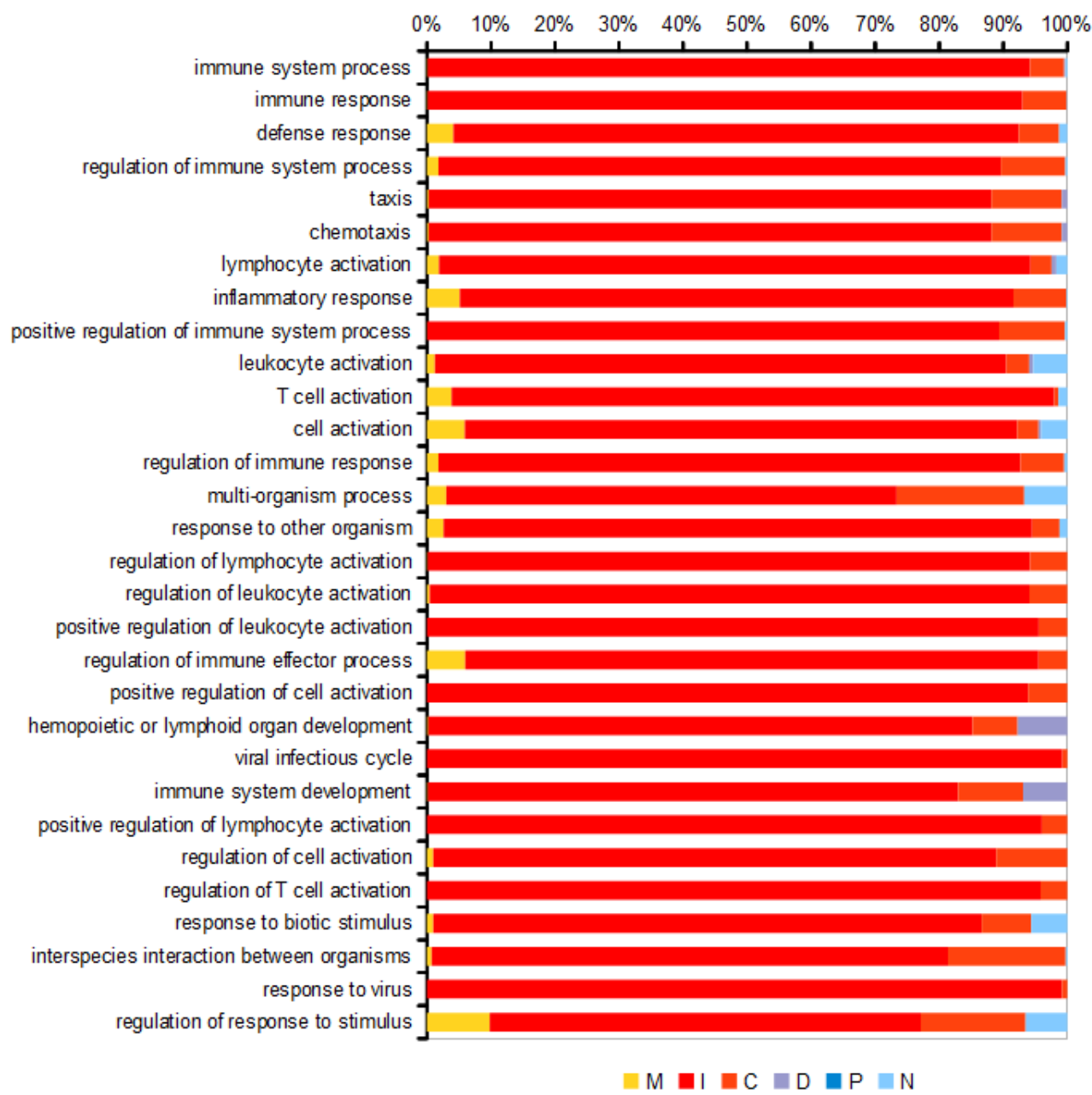

Cluster C

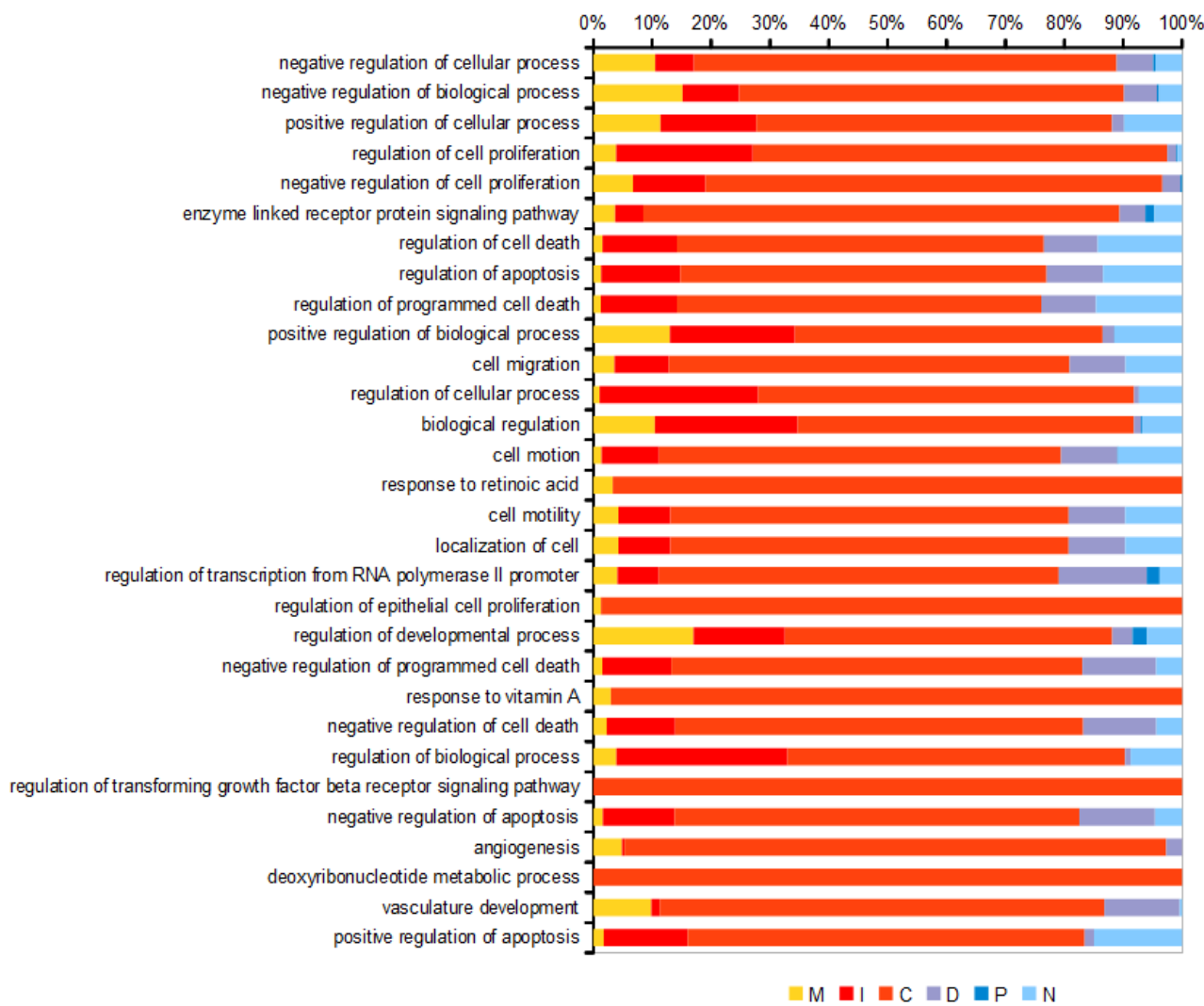

Cluster D

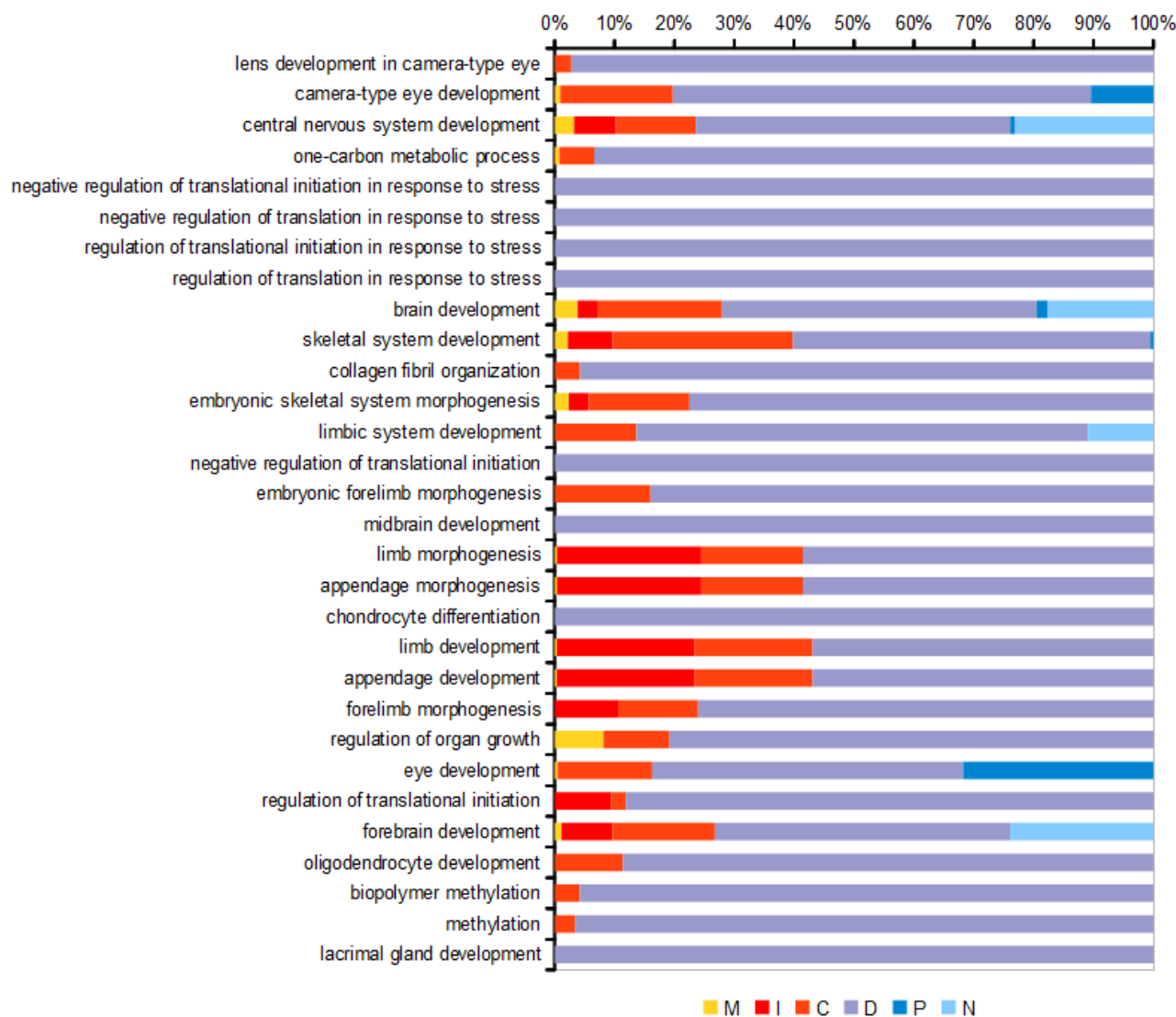

Cluster P

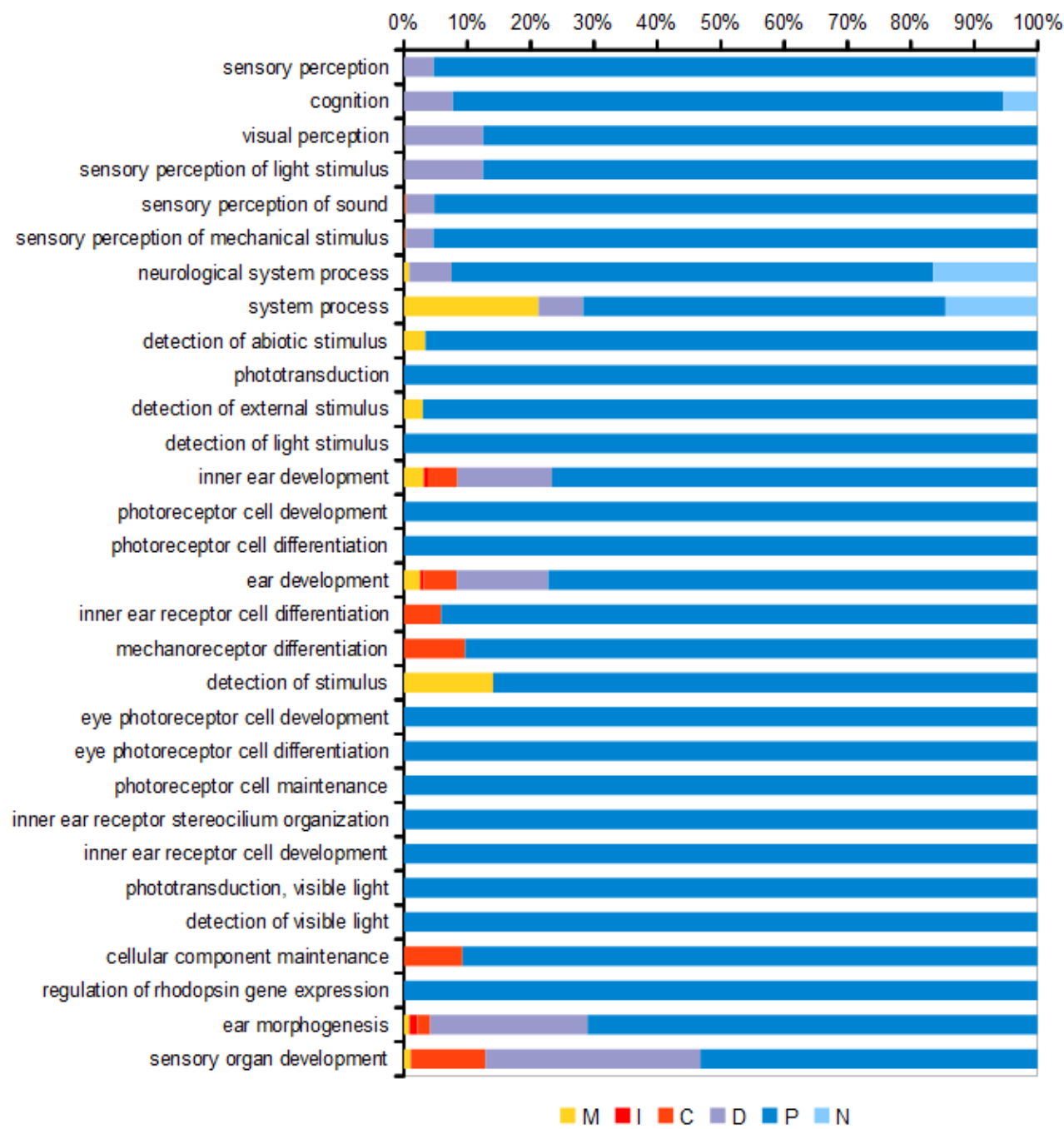

Cluster N

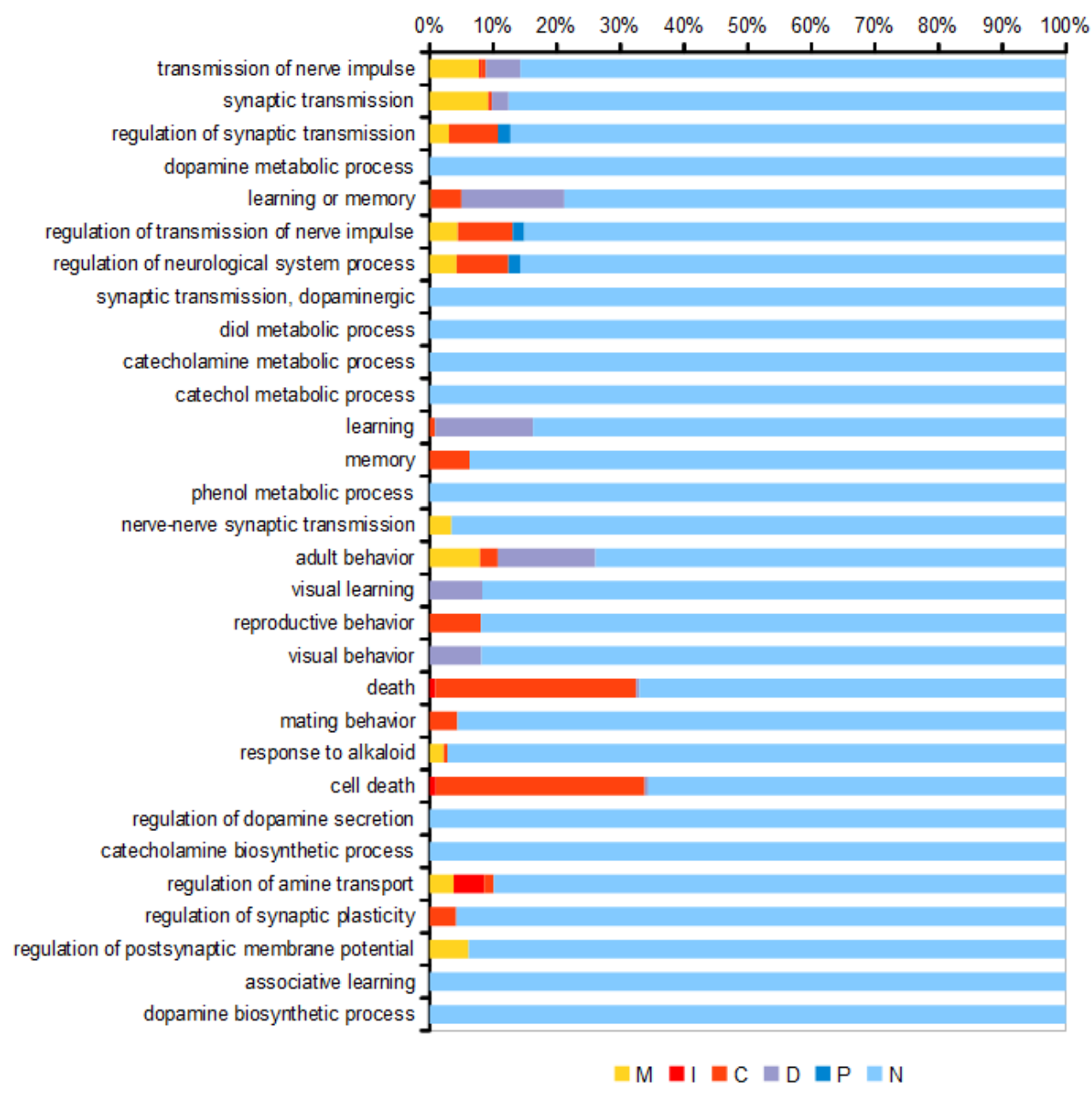

Supplement: Additional file 2 — Supplement 2. Comparison of GO biological processes among six disease clusters. [file 1752-0509-4-124-S2.PDF]

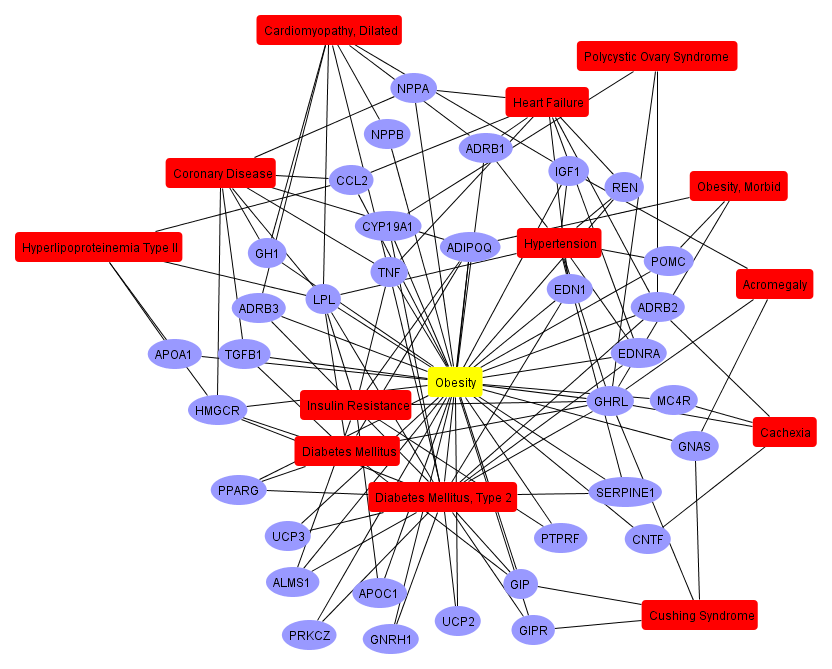

Supplement: Additional file 3 — Supplement 3. Disease network obtained for obesity and common causal genes. [file 1752-0509-4-124-S3.TIFF]

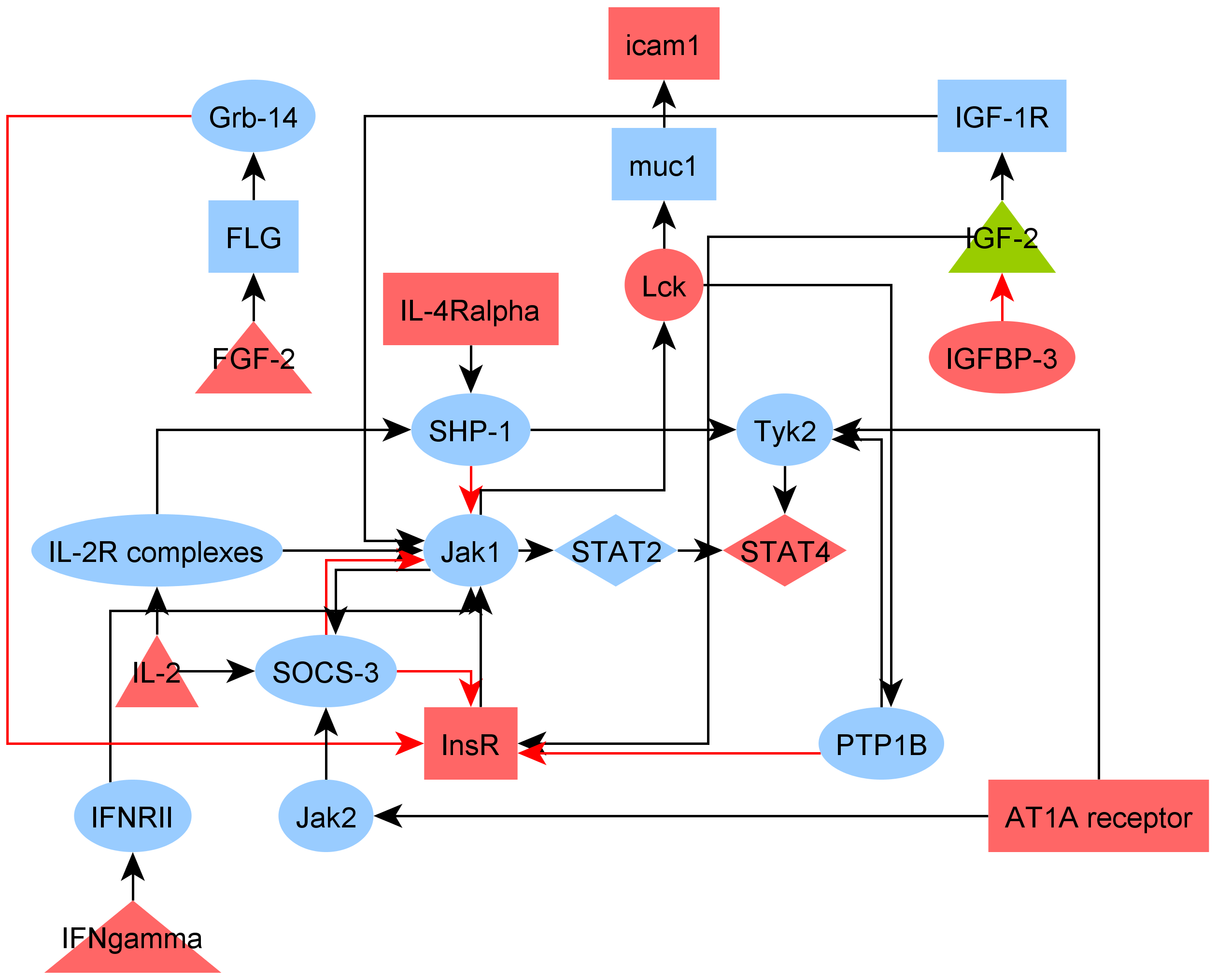

Supplement: Additional file 4 — Supplement 4. Network of causal components obtained for T1DM. [file 1752-0509-4-124-S4.TIFF]

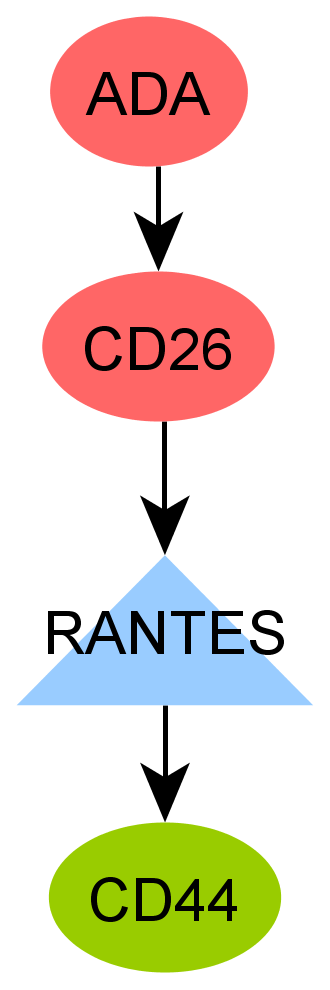

Supplement: Additional file 5 — Supplement 5. Small network of causal components obtained for T2DM. [file 1752-0509-4-124-S5.TIFF]

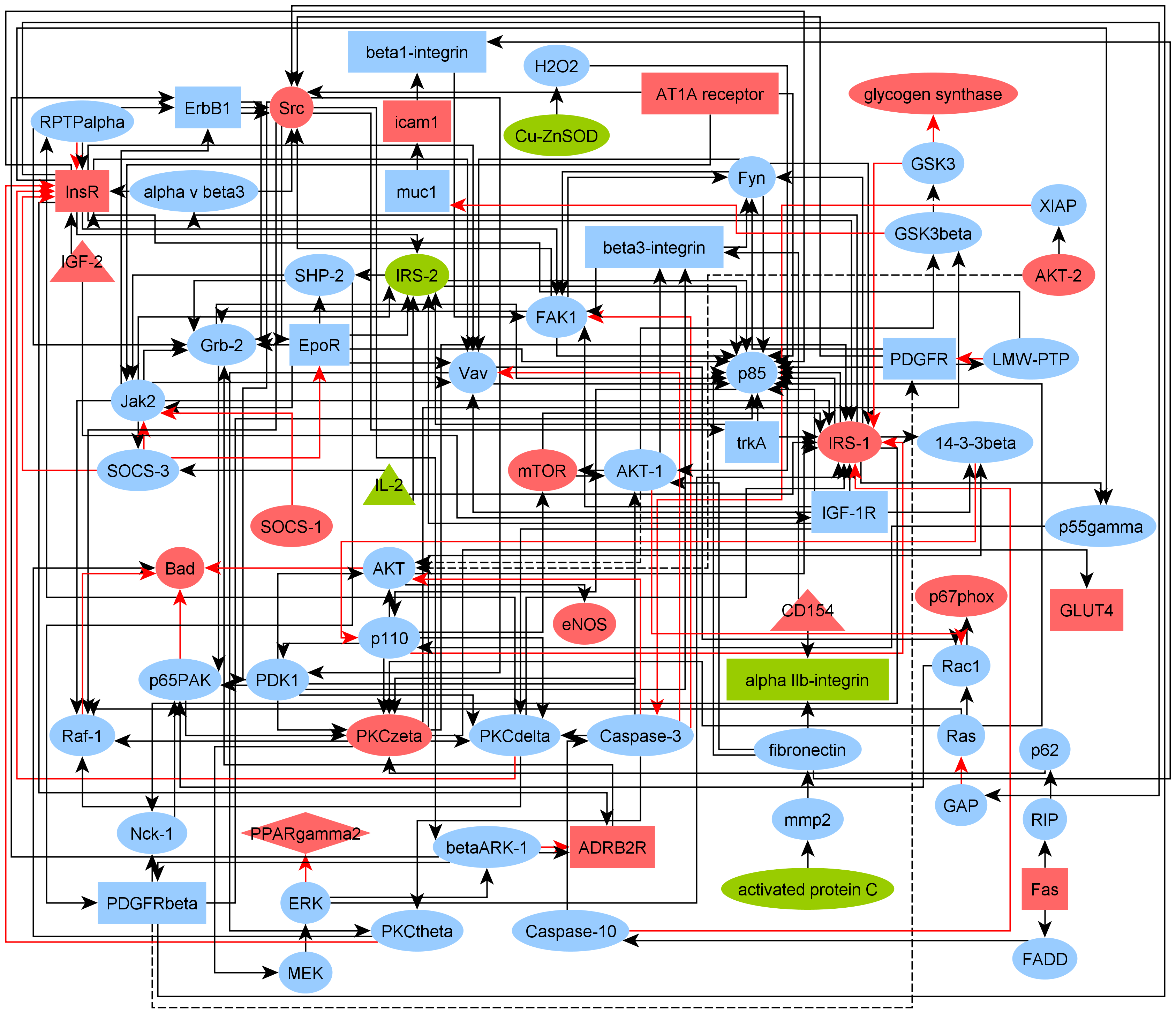

Supplement: Additional file 6 — Supplement 6. Large network of causal components obtained for T2DM. [file 1752-0509-4-124-S6.TIFF]
